# Radiation-induced brain injury: A review

**DOI:** 10.3389/fonc.2012.00073

**Published:** 2012-07-19

**Authors:** Dana Greene-Schloesser, Mike E. Robbins, Ann M. Peiffer, Edward G. Shaw, Kenneth T. Wheeler, Michael D. Chan

**Affiliations:** ^1^ Department of Radiation Oncology, Wake Forest School of Medicine,Winston-Salem, NC, USA; ^2^ Brain Tumor Center of Excellence, Wake Forest School of Medicine,Winston-Salem, NC, USA; ^3^ Department of Radiology, Wake Forest School of Medicine,Winston-Salem, NC, USA

**Keywords:** brain injury, hippocampal changes, metastatic brain tumor, pathogenesis, radiation-induced

## Abstract

Approximately 100,000 primary and metastatic brain tumor patients/year in the US survive long enough (>6 months) to experience radiation-induced brain injury. Prior to 1970, the human brain was thought to be highly radioresistant; the acute CNS syndrome occurs after single doses >30 Gy; white matter necrosis occurs at fractionated doses >60 Gy. Although white matter necrosis is uncommon with modern techniques, functional deficits, including progressive impairments in memory, attention, and executive function have become important, because they have profound effects on quality of life. Preclinical studies have provided valuable insights into the pathogenesis of radiation-induced cognitive impairment. Given its central role in memory and neurogenesis, the majority of these studies have focused on the hippocampus. Irradiating pediatric and young adult rodent brains leads to several hippocampal changes including neuroinflammation and a marked reduction in neurogenesis. These data have been interpreted to suggest that shielding the hippocampus will prevent clinical radiation-induced cognitive impairment. However, this interpretation may be overly simplistic. Studies using older rodents, that more closely match the adult human brain tumor population, indicate that, unlike pediatric and young adult rats, older rats fail to show a radiation-induced decrease in neurogenesis or a loss of mature neurons. Nevertheless, older rats still exhibit cognitive impairment. This occurs in the absence of demyelination and/or white matter necrosis similar to what is observed clinically, suggesting that more subtle molecular, cellular and/or microanatomic modifications are involved in this radiation-induced brain injury. Given that radiation-induced cognitive impairment likely reflects damage to both hippocampal- and non-hippocampal-dependent domains, there is a critical need to investigate the microanatomic and functional effects of radiation in various brain regions as well as their integration at clinically relevant doses and schedules. Recently developed techniques in neuroscience and neuroimaging provide not only an opportunity to accomplish this, but they also offer the opportunity to identify new biomarkers and new targets for interventions to prevent or ameliorate these late effects.

## RADIATION-INDUCED BRAIN INJURY

**FIGURE 1:**
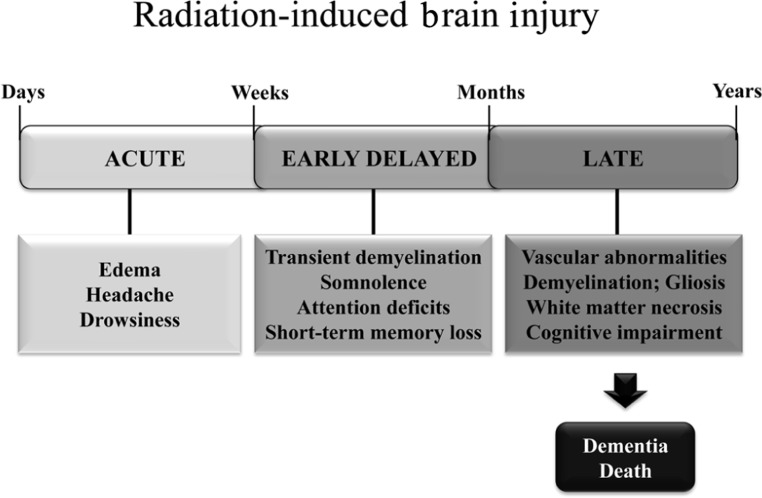
**Symptoms and timeline for the development of radiation-induced brain injury in patients treated with fWBI**.

Radiation-induced brain injury is often observed after fractionated partial or whole brain irradiation (fWBI); the syndrome includes both anatomic and functional deficits. Based on the time of clinical expression (**Figure 1**), radiation-induced brain injury is described in terms of acute, early delayed, and late delayed injury (Tofilon and Fike, 2000). Acute brain injury, expressed in days to weeks after irradiation, is rare with current radiation therapy techniques. Early delayed brain injury occurs 1–6 months post-irradiation and can involve transient demyelination with somnolence. Although both of these early injuries can result in severe reactions, they are normally reversible and resolve spontaneously. In contrast, late delayed brain injury, characterized histopathologically by vascular abnormalities, demyelination, and ultimately white matter necrosis (Schultheiss and Stephens, 1992), is usually observed >6 months post-irradiation; these late delayed injuries have been viewed as irreversible and progressive. Classically, late radiation-induced brain injury was viewed as due solely to a reduction in the proliferating capacity of glial (van den Maazen et al., 1993) or vascular endothelial (Calvo et al., 1988) cells. The loss of either of these cell types could ultimately produce white matter necrosis, but the loss of glial cells was thought to cause necrosis earlier than the loss of vascular endothelial cells. However, there is a growing awareness that patients receiving fWBI can have significant cognitive impairment at >6 months post-irradiation even when they do not have detectable anatomic abnormalities (Sundgren and Cao, 2009). The impact of cognitive impairment on a patient’s quality of life (QOL) is now recognized as second only to survival in clinical trials (Frost and Sloan, 2002).

## THE VASCULAR HYPOTHESIS OF LATE DELAYED RADIATION-INDUCED BRAIN INJURY

Proponents of the vascular hypothesis of late radiation-induced brain injury argue that vascular damage leads to ischemia and secondarily to white matter necrosis. In support of this hypothesis, a large amount of data has described radiation-induced vascular structural changes, including vessel wall thickening, vessel dilation, and endothelial cell nuclear enlargement (Calvo et al., 1988; Reinhold et al., 1990; Schultheiss and Stephens, 1992). Quantitative studies in irradiated rat brains have also demonstrated time- and dose-dependent reductions in the number of endothelial cell nuclei, blood vessel density, and blood vessel length (Reinhold et al., 1990; Brown et al., 2007). Moreover, white matter necrosis occurs in boron neutron capture studies where nearly all of the radiation damage is to the vasculature (Morris et al., 1996). A recent study in rodents has shown that capillary rarefaction and tissue hypoxia increased in all regions of the hippocampus 2 months after fWBI (Warrington et al., 2011a). Paradoxically, these investigators also showed that low ambient oxygen levels were able to restore the brain microvascular density (Warrington et al., 2011a,b) and reverse cognitive impairment (Warrington et al., 2012). Other studies have shown that (i) alterations of the blood–brain barrier (BBB) likely due to an imbalance in the levels of the matrix metalloproteinase-2 and the metalloproteinase-2 tissue inhibitor, (ii) degradation of collagen type IV, an extracellular matrix component of the blood vessel basement membrane (Lee et al., 2012), and (iii) changes in the mRNA and protein expression of VEGF, Ang-1, Tie-2, and Ang-2 (Lee et al., 2011) occur after clinically relevant single and fWBI doses. In a recent study, primary cultured mouse fetal neural stem cells injected into the tail vein after each of four 5 Gy fractions differentiated into both brain endothelial cells and a variety of brain cells; this restored the radiation-induced decrease in both cerebral blood flow and cognitive function (Joo et al., 2012). In contrast, radiation-induced necrosis has been reported in the absence of vascular changes (Schultheiss and Stephens, 1992). Also the PPARγ agonist, pioglitazone, and the ACE inhibitor, ramipril, that prevent radiation-induced cognitive impairment in the rat (Zhao et al., 2007a,b; Lee et al., 2012) do not reverse the reduction in vascular density and length that occurs after fWBI (Brown, unpublished data). Consequently, late radiation-induced brain injury cannot be solely due to vascular damage despite the large amount of evidence supporting this hypothesis.

## THE PARENCHYMAL HYPOTHESIS OF RADIATION-INDUCED BRAIN INJURY

### OLIGODENDROCYTES

The parenchymal hypothesis of radiation-induced brain injury initially focused on the oligodendrocyte that is required for the formation of myelin sheaths. The key cell for generating mature oligodendrocytes is the oligodendrocyte type-2 astrocyte (O-2A) progenitor cell that loses its reproductive capacity after WBI in the rat (Raff et al., 1983). It has been hypothesized that radiation-induced loss of O-2A progenitor cells leads to a failure to replace oligodendrocytes that ultimately results in demyelination and white matter necrosis. Although the oligodendrocyte population in young adult rats has been reported to be depleted within 24 h after single WBI doses of ≥3 Gy and total fWBI doses of ≥4.5 Gy (Bellinzona et al., 1996; Shinohara et al., 1997; Kurita et al., 2001), no change in the number of myelinated axons, the thickness of myelin sheaths, and the cross-sectional area of myelinated axons has been measured in cognitively impaired rats 12 months after a total fWBI dose of 40 Gy delivered twice a week for 4 weeks to middle-aged rats (Shi et al., 2009). Further, although the kinetics of oligodendrocyte depletion is consistent with an early transient demyelination, it is inconsistent with the late onset of white matter necrosis (Hornsey et al., 1981). Thus, the relationship between radiation damage to oligodendrocytes and late radiation-induced brain injury is still unclear.

### ASTROCYTES

These cells constitute approximately 50% of the total glial cell population in the brain and outnumber the neurons four to one in higher mammals (Hansson, 1988). Once viewed as playing a mere supportive role, astrocytes are now recognized as a heterogeneous class of cells that perform diverse functions, including modulation of synaptic transmission and secretion of neurotrophic factors such as basic fibroblast growth factor to promote neurogenesis (Song et al., 2002; Seth and Koul, 2008). Astrocytes have been shown to protect endothelial cells and neurons from oxidative injury (Wilson, 1997). Also, juxtacrine signaling between astrocytes and endothelial cells is critical for generation and maintenance of a functional BBB, the vascular structure that restricts entry of blood-borne elements into the brain (Janzer and Raff, 1987). In response to injury, astrocytes undergo proliferation, exhibit hypertrophic nuclei/cell bodies, and show increased expression of glial fibrillary acidic protein (GFAP; Seifert et al., 2006; Yuan et al., 2006; Seth and Koul, 2008; Wilson et al., 2009; Zhou et al., 2011). These reactive astrocytes secrete a host of pro-inflammatory mediators such as cyclooxygenase (Cox)-2 and the intercellular adhesion molecule (ICAM)-1, which may aid the infiltration of leukocytes into the brain via BBB breakdown (Kyrkanides et al., 1999; Yuan et al., 2006; Wilson et al., 2009; Zhou et al., 2011). Irradiating the rat and mouse brain increases GFAP protein levels, both acutely (24 h) and chronically (4–5 months; Chiang et al., 1993; Hong et al., 1995). Conditioned medium from irradiated microglial cells has been shown to induce astrogliosis which might contribute to radiation-induced edema (Hwang et al., 2006). However, the exact role of astrocytes in the overall pathogenesis of late radiation-induced brain injury is still unclear, but they likely contribute by interacting with both vascular and other parenchymal elements in the brain.

### MICROGLIA

These immune cells represent about 12% of the total brain cells (Gebicke-Haerter, 2001). In an uninjured brain, microglia actively monitor the microenvironment to ensure that homeostasis is maintained (Stoll and Jander, 1999). Microglia express neurotrophins that selectively regulate (i) microglial function, (ii) secretion of neurotrophic factors which promote neuronal survival, and (iii) proliferation (Elkabes et al., 1996). After injury, microglia become activated, a process characterized by rounding of the cell body, retraction of cell processes, proliferation, and increased production of reactive oxygen species (ROS), cytokines, and chemokines that mediate neuroinflammation (Stoll and Jander, 1999; Gebicke-Haerter, 2001; Pocock and Liddle, 2001; Kim and de Vellis, 2005).

Although microglial activation plays an important role in phagocytosis of dead cells, sustained activation is thought to contribute to a chronic inflammatory state in the brain (Gebicke-Haerter, 2001; Joo et al., 2012). Tissue culture studies have demonstrated that irradiating activated microglia leads to a marked increase in expression of the pro-inflammatory genes TNFα, IL-1β, IL-6, and Cox-2, and the chemokines, MCP-1 and ICAM-1 (Chiang et al., 1993; Kyrkanides et al., 1999, 2002; Hwang et al., 2006; Lee et al., 2010). Rodent studies have also detected (i) an increase in pro-inflammatory mediators within hours after irradiating the brain (Chiang et al., 1997; Kyrkanides et al., 2002; Lee et al., 2010), and (ii) an increase in the percentage of activated microglia in the brain during the latent period before the expression of late radiation-induced brain injury (Mildenberger et al., 1990; Chiang et al., 1997; Monje et al., 2003). Rodent studies and analysis of human brain tissue also suggest that microglial activation may be associated with decreased hippocampal neurogenesis and cognitive function (Monje et al., 2002, 2007; Raber et al., 2004). Anti-inflammatory agents such as ramipril and indomethacin reduce the number of activated microglia in the hippocampus and/or perirhinal cortex and prevent radiation-induced cognitive impairment in rodents (Monje et al., 2003; Lee et al., 2012). However, the anti-inflammatory agent, L-158, 809, has no effect on microglial activation, but still prevents radiation-induced cognitive impairment (Robbins et al., 2009; Conner et al., 2010). Finally, orthotopic injections of fetal neuronal stem cells (NSC) that form new neurons without affecting the number of activated microglia reverse radiation-induced cognitive impairment in rodents (Acharya et al., 2009, 2011). Thus, the exact role that activated microglia play in generating radiation-induced brain injury, including cognitive impairment, is still an open question.

### NEURONS

Once considered a radioresistant population because they no longer could divide, neurons have now been shown to respond negatively to radiation. Studies have demonstrated radiation-induced changes in hippocampal cellular activity (Gangloff and Haley, 1960; Bassant and Court, 1978), synaptic efficiency/spike generation (Bassant and Court, 1978; Pellmar and Lepinski, 1993), and neuronal gene expression (Noel et al., 1998; Rosi et al., 2008). For example, irradiating the rodent brain with single and fractionated doses produces changes in (i) neuronal receptor expression of the immediate-early gene activity-regulated cytoskeleton-associated protein (Arc) (Rosi et al., 2008), (ii) *N*-methyl-D-aspartic acid (NMDA) receptor subunits (Shi et al., 2006; Machida et al., 2010), (iii) glutaminergic transmission (Rohde et al., 1979; Machida et al., 2010), and (iv) hippocampal long-term potentiation (LTP; Snyder et al., 2001; Vlkolinsky et al., 2008); all are important for synaptic plasticity and cognition. Interestingly, these changes can occur in the absence of alterations in the total number of mature neurons, the number of myelinated axons, the thickness of myelin sheaths, and/or the cross-sectional area of myelinated axons following fWBI (Shi et al., 2008). Thus, subtle cellular and/or molecular changes in the neurons themselves or subtle changes in the association/communication between neurons and astrocytes must play an as yet unidentified role in late radiation-induced cognitive impairment.

## THE DYNAMIC INTERACTIONS BETWEEN MULTIPLE CELL TYPES HYPOTHESIS

Because no single cell or tissue associated with either the vascular or parenchymal hypotheses can fully explain late delayed radiation-induced brain injury, including cognitive impairment, radiation-induced late effects are now hypothesized to occur due to dynamic interactions between the multiple cell types in the brain (Tofilon and Fike, 2000). Vascular endothelial cells, oligodendrocytes, astrocytes, microglia, and neurons, are now viewed not as passive bystanders that merely die from radiation damage, but rather as active participants in an orchestrated response to radiation injury that, theoretically, allows one to change the response/outcome by intervening at numerous points in the process to prevent or ameliorate the development of late radiation-induced brain injury, including cognitive impairment. It is likely that the successful unraveling of this puzzle will require the detection of subtle molecular, cellular, and microanatomic changes in the brain that will clearly challenge basic science and clinical investigators over the next decade.

## COGNITIVE IMPAIRMENT IN BRAIN TUMOR SURVIVORS AFTER fWBI

**FIGURE 2:**
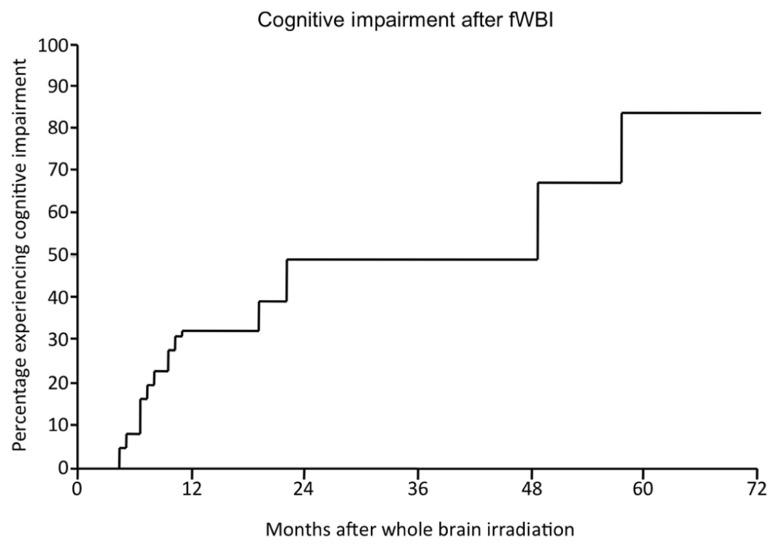
**The percentage of patients developing radiation-induced cognitive impairment as a function of time after fWBI.** Adapted from Nieder et al. (1999).

Radiation-induced cognitive impairment, including dementia, is reported to occur in up to 50–90% of adult brain tumor patients who survive >6 months post-irradiation (Crossen et al., 1994; Giovagnoli and Boiardi, 1994; Johannesen et al., 2003; Meyers and Brown, 2006). This cognitive impairment is marked by decreased verbal memory, spatial memory, attention, and novel problem-solving ability (Hochberg and Slotnick, 1980; Twijnstra et al., 1987; Laukkanen et al., 1988; Roman and Sperduto, 1995). Nieder et al. (1999) described significant cognitive impairment in 49% of patients at 2 years after treatment with fWBI; the incidence and severity continued to rise over time (**Figure 2**). Chang et al. (2009) documented a detectable cognitive impairment at 4 months after fWBI compared to patients treated with radiosurgery. Moreover, radiation-induced cognitive impairment occasionally progresses to dementia where patients experience progressive memory loss, ataxia, and urinary incontinence (Vigliani et al., 1999). Radiation-induced dementia is a rare occurrence with fraction sizes <3 Gy (DeAngelis et al., 1989; Klein et al., 2002). However, patients who survive >2 years after fWBI have a continually increasing risk of developing dementia over time (Scott et al., 1999). Importantly, all of these late sequelae can be seen in the absence of radiographic or clinical evidence of demyelination or white matter necrosis (Dropcho, 1991; Shaw et al., 2006).

In spite of the relative rarity of progressing to frank dementia, radiation-induced cognitive impairment has significant effects on QOL. The majority of >6 month survivors of partial or whole brain irradiation have a symptom cluster consisting of fatigue, changes in mood, and cognitive dysfunction (Gleason et al, 2007). Results of neurocognitive testing in a phase III clinical trial (PCI P120-9801) showed a significant correlation between performance on the Functional Assessment of Cancer Therapy-Brain Specific (FACT-Br) test and a patient’s QOL as measured by the ability to perform daily living activities (Li et al., 2008). In the Nieder et al. (1999) study, 20% of patients treated with fWBI had a >10% decline in Karnofsky Performance Status due to radiation-induced cognitive impairment. Furthermore, brain tumor patients are surviving longer due to improved radiation therapy techniques and systemic therapies (Stupp et al., 2005; Cochran et al., 2012), so the patient population experiencing radiation-induced cognitive impairment is growing rapidly. Consequently, the search for (i) biomarkers to identify patients who will/will not develop cognitive impairment after fWBI and (ii) therapeutic strategies to prevent/ameliorate radiation-induced cognitive impairment have become very important.

## ASSESSING RADIATION-INDUCED COGNITIVE IMPAIRMENT IN THE CLINIC

The assessment of radiation-induced cognitive impairment in the clinic has evolved over time. The mini-mental status examination (MMSE), a test for global cognitive function which has been validated in other cognitive disorders, is relatively insensitive for assessing radiation-induced cognitive impairment (Herman et al., 2003; Kondziolka et al., 2005). The MMSE (i) does not avoid memorized learning from repeat testing, (ii) is biased against patients with lower educational backgrounds, and (iii) is relatively insensitive to the subtle changes in function caused by brain radiotherapy. To overcome these problems, recent cognitive assessments have focused on the specific domains that are most affected by brain irradiation (Klein et al., 2002; Shaw et al., 2006; Chang et al., 2009).

Using intense neurocognitive assessments on primary and metastatic brain tumor patients has been criticized because many of their tumors recur leading to a general decline in health and death. As such, patients are generally less willing to participate in intense cognitive testing as their health deteriorates, and the utility of the results from those that do participate is questionable. Recently, RTOG study formulated a battery of tests that focuses on the cognitive domains known to be affected by brain irradiation, including memory, verbal fluency, visual motor speed, and executive function (**Table 1**); the estimated time of completion is ~30 min. In recent trials, this battery of cognitive tests appears to overcome this major obstacle to assessing radiation-induced cognitive impairment in brain tumor patients.

**Table 1:** Neurocognitive batteries used in modern prospective clinical trials.

Trial	Intelligence	Perception/psychomotor speed	Memory	Attention/executive function
EORTC	Dutch adult reading test	Line bisection test	Working memory task	Stroop color word test
		Facial recognition test	Visual verbal learning test	Categoric word fluency test
		Judgment of line orientation		Concept shifting test
		Letter-digit substitution		
RTOG 0614	COWA	Trail-making A	Hopkins verbal learning test	Trail-making B
RTOG 0933	N/A	N/A	Hopkins verbal learning test	N/A
			One card learning test	
			International shopping list test	
MDACC	N/A	N/A	Hopkins verbal learning test	N/A
CCOP 97100	COWA	Trail-making A	California verbal learning test	Trail-making B
			Rey Osterrieth complex figure	
			Digit span	

## EVALUATION OF PATIENT POPULATIONS FOR STUDYING RADIATION-INDUCED COGNITIVE IMPAIRMENT

Several patient populations have been used to study radiation-induced cognitive impairment. These populations include (i) patients receiving prophylactic cranial irradiation (PCI) (Twijnstra et al., 1987; Laukkanen et al., 1988; Grosshans et al., 2008), (ii) patients with nasopharyngeal cancer (Cheung et al., 2000; Hsiao et al., 2010), (iii) patients with low-grade gliomas (Taphoorn et al., 1994; Klein et al., 2002), (iv) patients with benign non-parenchymal brain tumors (Gondi et al., 2011), and (v) patients with primary (Klein et al., 2002) or metastatic brain tumors (Nieder et al., 1999). The majority of these patients have (i)primary brain tumors treated with temozolomide and a variety of radiation therapy techniques or (ii) metastatic brain tumors treated with fWBI or radiosurgery. In general, about 50–70% of these patients survive long enough (>6 months) to develop radiation-induced cognitive impairment that affects their QOL. Therefore, this is the population that presents the greatest challenge to the radiation oncologist. Nevertheless, it is also the population with the greatest number of confounding factors (e.g., short life spans with declining health, tumor effects on brain regions associated with cognition, prior treatment of systemic disease with a variety of chemotherapeutic agents, concurrent treatment with chemotherapy, steroids, and neurotrophic drugs) that, by themselves, can affect cognition. Therefore, studying populations, who receive fWBI but do not have fast growing tumors in the brain, could provide important data on the role that radiation damage plays in generating cognitive impairment in primary and metastatic brain tumor patients.

### SMALL CELL LUNG CANCER PATIENTS

The NCI published a study on 15 SCLC patients who were long-term survivors after PCI and found that 12 of these exhibited abnormalities on neuropsychiatric testing, while seven performed below the normal range on the MMSE test (Johnson et al., 1990). However, in a larger study of 69 SCLC patients who received PCI, a substantial portion of the patients exhibited cognitive impairments prior to PCI, and multivariate analysis could not identify any significant cognitive differences before and after PCI (Grosshans et al., 2008). Finally, in another recent study, patients who received PCI had a detectable decline in verbal memory just 6–8 weeks after completion of PCI (Welzel et al., 2008). Thus, the radiation-induced cognitive impairment data from SCLC patients who receive PCI is confusing at best, probably because these patients received chemotherapy and/or larger radiation fractions that are not typical of those used to treat primary and metastatic brain tumor patients.

### NASOPHARYNGEAL CANCER PATIENTS

Survivors of nasopharyngeal cancer offer another opportunity to measure radiation-induced cognitive impairment in the absence of a brain tumor. Patients treated for nasopharyngeal cancer routinely have high doses of radiation delivered to the bilateral temporal lobes because of the need to treat the superior retropharyngeal lymph nodes. These patients have ~70% chance of long-term survival, and thus, the potential for development of radiation-induced cognitive impairment, primarily due to damage to the temporal lobes. Cheung et al. (2000) reported that temporal lobe necrosis predicted a worsening of cognitive impairment in 50 irradiated nasopharyngeal cancer patients who were followed longitudinally with neuropsychological testing. Recently, Hsiao et al. (2010) demonstrated that nasopharyngeal cancer patients treated with intensity-modulated radiotherapy (IMRT) had a worse cognitive outcome if >10% of their temporal lobe volume received a total fractionated dose of >60 Gy than patients who received <60 Gy.

### LOW-GRADE GLIOMA PATIENTS

In a seminal publication by Klein et al. (2002), cognitive outcomes of patients with low-grade glioma were compared to both patients with indolent lymphomas that had no CNS disease and healthy controls. The radiotherapy fields used in this study generally did not include the entire brain. This analysis revealed that low-grade gliomas, anti-epileptic medications, and radiotherapy could each produce cognitive impairment; cognition was most affected if fractions >2 Gy were used. Consequently, radiation-induced cognitive data from low-grade glioma patients are also not likely to provide information relevant to the majority of primary and metastatic brain tumor patients. Shaw et al published results of a randomized trial in 200 adult low-grade glioma patients who received either 50.4 Gy or 64.8 Gy at 1.8 Gy per fraction to partial brain treatment fields (Shaw etal, 2002). This is the only known modern primary brain tumor study in which patients were randomized to receive low- versus high dose-radiation. There were no differences in survival outcomes by dose. However, the incidence of radiation necrosis (i.e., grade 3, 4 or 5 late brain toxicity) However, the 5-year actuarial incidence of radiation necrosis (i.e., grade 3, 4 or 5 late brain toxicity) was 10% in patients receiving 64.8 Gy versus 5% for those given 50.4 Gy.

### BENIGN NON-PARENCHYMAL BRAIN TUMOR PATIENTS

Arguably, the ideal populations for determining the radiation tolerance of various brain regions are the patients with benign non-parenchymal brain tumors such as meningiomas, pituitary tumors, and schwannomas. These tumors generally do not affect cognition and are not treated with chemotherapy. Patients with these tumors have life expectancies long enough after fWBI to experience radiation-induced cognitive impairment. Finally, the results of these human studies could be compared to the results of preclinical animal studies on radiation-induced brain injury, including cognitive impairment, all of which have been performed in animals that have no brain tumors or neurological diseases (Lamproglou et al., 1995; Yoneoka et al., 1999; Raber et al., 2004; Rola et al., 2004). Such a comparison could greatly facilitate the development of molecular, cellular, or imaging biomarkers of the onset and progression of radiation-induced cognitive impairment or interventions that could be successfully translated to the clinic. Presently, the only published report on patients with benign non-parenchymal brain tumors indicates that avoiding or lowering the dose to the hippocampus will reduce radiation-induced cognitive impairment (Gondi et al., 2011); the equivalent study has not been performed in animals.

From the above discussion, it is distinctly possible that the molecular, cellular, and microanatomic events that lead to radiation-induced cognitive impairment are different for SCLC, nasopharyngeal cancer, low-grade glioma, benign non-parenchymal brain tumor, primary brain tumor, and metastatic brain tumor patients. Consequently, (i) identifying biomarkers of the onset and progression of radiation-induced cognitive impairment and (ii) developing therapeutic strategies to prevent or ameliorate radiation-induced cognitive impairment is likely to be challenging for both basic scientists and physicians.

## THE NEUROANATOMICAL TARGET THEORY OF RADIATION-INDUCED COGNITIVE IMPAIRMENT

The target structures and dose thresholds for the development of radiation-induced cognitive impairment are of current clinical interest. Prior studies have suggested that partial brain irradiation may not cause the same degree of cognitive impairment as WBI (Armstrong et al., 1995; Torres et al., 2003). This observation could be explained by hypothesizing that there are specific brain regions that lead to cognitive impairment. When the entire brain is irradiated, no structure will be spared that could provide some normal or compensatory cognitive function. A recent dose-volume histogram analysis of two prospective clinical trials by Leyrer et al. (2011) indicates that it is not the dose to the whole brain, but rather the dose to the hippocampus and temporal lobes that predicts the subsequent radiation-induced cognitive impairment. These authors proposed a neuroanatomical target theory, which suggests that selective damage to certain brain structures may be the cause of cognitive impairment after radiotherapy. A corollary of such a theory is that selective avoidance of these brain structures may be able to preserve cognitive function. Recent advances in radiation therapy planning, including the advent of stereotactic localization (Shrieve et al., 1994), image guidance (Gutiérrez et al., 2007), IMRT (Barani et al., 2007b; Gutiérrez et al., 2007), and proton beam radiotherapy (Rosenschold et al., 2011) have made it possible to selectively avoid brain structures such as the hippocampus and temporal lobes to test this theory.

## NON-INVASIVE IMAGING BIOMARKERS OF RADIATION-INDUCED COGNITIVE IMPAIRMENT

Currently, there are no validated biomarkers for determining who will/will not develop radiation-induced brain injury, including cognitive impairment, or who will/will not respond favorably to therapies aimed at preventing or ameliorating these cognitive deficits. Radiation-induced late effects in the brain occur within the closed cranial cavity. Therefore, non-invasive techniques are needed to study this significant side effect of brain tumor radiotherapy. Given that radiation-induced cognitive impairment can occur in the absence of radiographic evidence of gross anatomical changes, X-ray computed tomography (CT), T1/T2 magnetic resonance imaging (MRI), and ultrasound techniques are not likely to provide information relevant to the onset and progression of radiation-induced cognitive impairment. However, both MRI and positron emission tomography (PET) have the ability to interrogate metabolic, physiologic, and functional properties of the brain. MRI utilizes magnetic fields to generate information by exciting the protons in hydrogen atoms and monitoring them as they relax. Depending on the pulse sequence, differences in the magnetic susceptibility properties of tissues can be exploited to probe various molecular, cellular, microanatomic, and physiologic properties of normal and tumor tissues. Magnetic resonance spectroscopy (MRS) utilizes an MR scanner to identify and quantify metabolites that reflect both the cellular properties and environmental conditions in specific regions of normal and tumor tissues. PET utilizes radioligands that contain an atom that emits a positron to interrogate the metabolic, receptor, physiologic, and functional properties of normal and tumor tissue. Theoretically, these three non-invasive techniques have the ability to identify biomarkers of the onset and progression of radiation-induced cognitive impairment.

## NON-INVASIVE VASCULAR BIOMARKERS OF RADIATION-INDUCED COGNITIVE IMPAIRMENT

Vascular injury has been hypothesized to play a critical role in the development of late radiation-induced injury, including radiation necrosis (Brown et al., 2005; Yuan et al., 2006). Shortly after fWBI, vascular structure and function can be altered; these alterations include blood vessel dilatation, endothelial cell enlargement, capillary loss, and perivascular astrocyte hypertrophy which can lead to BBB disruption, increased permeability, and edema. This acute vascular injury has the potential to be detectable by MRI prior to the development of radiation-induced demyelination and white matter necrosis (Reinhold et al., 1990; Li et al., 2003).

Dynamic contrast-enhanced (DCE) MRI uses T1-weighted imaging to quantitatively assess vascular permeability by repeatedly imaging the brain prior to and following a bolus i.v. injection of a gadolinium contrast agent. By tracking the movement of the contrast agent through the brain as a function of post-injection time, and calculating the transfer constant, *K*^trans^, using a compartment model that describes the kinetics, the passive leakage of the contrast agent from the intravascular to the extravascular extracellular space can be obtained (Tofts et al., 1999). High *K*^trans^ values indicate that the BBB is not intact; low *K*^trans^ values indicate that the BBB is intact. It has been suggested that these increases in the BBB permeability after fWBI are the result of vascular endothelial cell death (Li et al., 2003). Presently, there is no evidence that DCE measured changes in BBB permeability are a biomarker for the onset or progression of late radiation-induced cognitive impairment.

Functional MRI (fMRI) measures the oxyhemoglobin to deoxyhemoglobin ratio in the brain to obtain an estimate of blood flow. Oxyhemoglobin is diamagnetic and does not generate an MR signal; deoxyhemoglobin is paramagnetic and emits a relatively strong MR signal. If a brain region of interest (ROI) is actively involved in a task, the area uses more oxygen, so the deoxyhemoglobin level in the ROI increases. This increase in deoxyhemoglobin generates an increased MR signal, but the signal is out of phase with the normal brain signal, and thus, appears as a decrease in the T2-weighted brain signal due to phase interference. This decrease in the MR signal is called the blood oxygenation level-dependent (BOLD) signal.

In a small study of childhood cancer survivors (*n* = 16), fMRI was used to compare the activity in the visual cortex of childhood survivors, unirradiated siblings, and unirradiated adults during a visual task (Zou et al., 2005). Overall the timing of the BOLD signal triggered by the visual event was the same across all groups. However, the BOLD signal decreased in the childhood cancer survivors to a value less than the baseline and stayed there for a prolonged time before recovering. The survivors also had an overall reduction in the BOLD signal in the visual cortex when compared to unirradiated siblings and adults. The number of voxels that had an increase in the BOLD signal was greatest for those receiving irradiation to both the brain and spinal cord. However, there was no difference in the number of voxels that had an increase in BOLD signal between those treated with chemotherapy and those that were not. No similar study has been undertaken with either adults or using a cognitive task. To date, we are unaware of a BOLD study in unanesthetized pediatric or adult animal models. Consequently, there is no direct evidence at this time that fMRI is likely to identify a non-invasive biomarker of radiation-induced cognitive impairment.

Arterial spin labeling (ASL) involves placing a pulsed or continuous RF field on the carotid artery in the neck to align the spins of the water protons in the blood (Detre et al., 2009). When the blood leaves the RF field, the proton spins return to their normal state producing an MR signal. The difference between the brain MR signal, with and without the RF field on, can be used to calculate the blood flow in a specific brain region before and after fWBI. Increases or decreases in blood flow are interpreted as increases or decreases in the activity or function of a specific brain region. By determining the blood flow in various regions associated with cognition before and after fWBI, it may be possible to obtain a non-invasive biomarker that predicts the onset and/or progression of radiation-induced cognitive impairment. However, there are no reports of a correlation between blood flow determined by ASL and radiation-induced cognitive impairment at this time.

## NON-INVASIVE PARENCHYMAL BIOMARKERS OF RADIATION-INDUCED COGNITIVE IMPAIRMENT

Proton MRS is a non-invasive technique that uses an MR scanner to (i) identify and quantify metabolites in the brain (Hoehn et al., 2001; Gillies and Morse, 2005), (ii) differentiate radiation necrosis from brain tumor progression (Chong et al., 2002; Schlemmer et al., 2002), and (iii) serve as a indicator of neurotoxicity following experimental (Yousem et al., 1992; Herynek et al., 2004) and clinical brain irradiation (Esteve et al., 1998; Walecki et al., 1999; Virta et al., 2000; Chan et al., 2001; Lee et al., 2004; Sundgren et al., 2009). Brain metabolites that have been quantified include choline/phosphocholine (Cho/pCho), creatine/phosphocreatine (Cr/pCr), glutamate (Glu), glutamine (Gln), *N*-acetyl-aspartate (NAA), myoinositol (mI), taurine (tau), and lactate. The concentration of each of these metabolites can be quantified in voxels as small as ~15 mm^3^ in the rodent brain (Shi et al., 2011) with a 7T MR scanner and ~0.7 cm^3^ in humans (Robbins et al., 2012) with a 3T MR scanner. NAA and Glu are predominantly neuronal markers; changes in their concentrations have been associated with neuronal damage after fWBI (Shi et al., 2011) or neurological diseases such as Alzheimer’s (Kaiser et al., 2005; den Heijer et al., 2006). Gln and mI are predominantly glial cell markers; changes in their concentrations have been associated with glial damage after fWBI (Pasantes-Morales et al., 2000; Shi et al., 2011). Cho/pCho is associated with cell membrane synthesis; concentration changes are associated with changes in cell proliferation and inflammatory cell infiltration (Robbins et al., 2012). Cr/pCr is a marker of energy metabolism; its concentration is relatively constant throughout the brain before and after fWBI (Sundgren and Cao, 2009).

**FIGURE 3:**
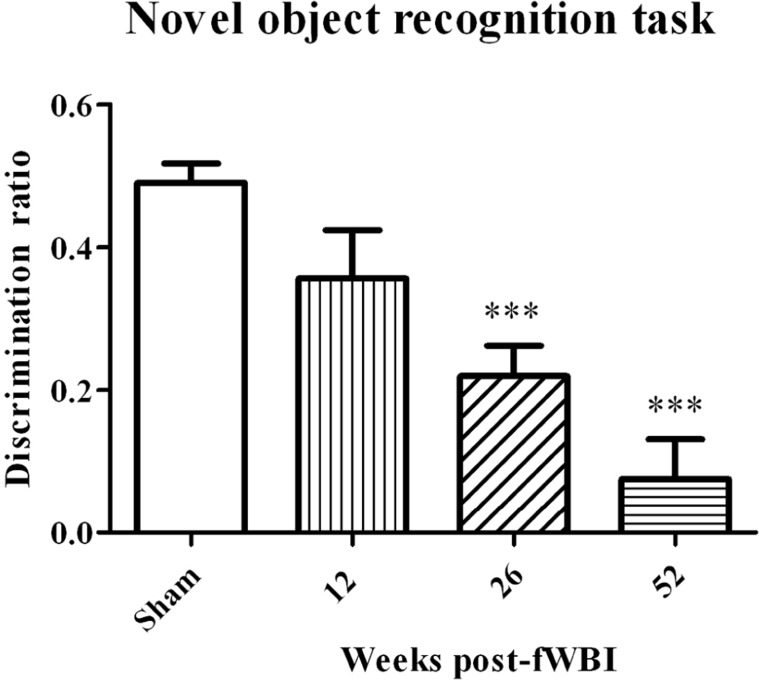
**Development of radiation-induced cognitive impairment as a function of time after young adult male Fischer 344 X Brown Norway rats were irradiated with a total 40 Gy dose of fWBI delivered as 5 Gy fractions, twice/week for 4 weeks.** Cognition was assessed using the novel object recognition (NOR) task. The sham-irradiated group value is the average of the NOR scores from unirradiated rats at all of the time points. In this rat model, cognitive impairment is both progressive and not significantly different from sham-irradiated rats until ~6 months after fWBI, similar to what is observed in the clinic. ***P <0.001.

Very little preclinical data are available on MRS detection of metabolite changes in the normal brain following irradiation. Using a 4.7T MR scanner, Herynek et al. (2004) observed decreases in Cr and NAA at 8 and 12 months after bilateral Gamma Knife irradiation with a dose of 35 Gy to the hippocampus of young adult male rats; this dose resulted in severe functional and structural brain damage. Chan et al. (2009) used a 7T MR scanner to determine significant increases in Cho, Glu, tau, and lactate levels at 12 months after the right half of young adult male rat brains were irradiated with a single 28 Gy dose of 6 MV photons. These changes in white matter were confirmed histologically at postmortem. Finally, Atwood et al. (2007) used a 7T MR scanner to demonstrate a potential relationship between radiation-induced changes in NAA/tCr, Glu + Gln/tCr, and mI/tCr concentrations in the rat brain after a 40 Gy total dose delivered in 5 Gy fractions, twice per week for 4 weeks and cognitive impairment measured by the novel object recognition test at 12 months after fWBI. However, additional experiments using this rat model of progressive radiation-induced cognitive impairment (**Figure 3**) demonstrated that cognitive impairment occurred before changes in these brain metabolites (Robbins et al., 2009). Thus, none of the brain metabolite changes could serve as a biomarker (i) for the onset/progression of radiation-induced cognitive impairment or (ii) to assess the response to interventions that might prevent/ameliorate radiation-induced cognitive impairment in this rat model.

Clinically, MRS has been used to assess metabolite changes in normal appearing white matter after fWBI (Esteve et al., 1998; Walecki et al., 1999; Virta et al., 2000; Lee et al., 2004; Sundgren et al., 2009). Acute lymphoblastic leukemia survivors, treated with intrathecal methotrexate and PCI, had decreasing NAA:Cr and Cho:Cr ratios as a function of time (5.6–19 years) after fWBI (Chan et al., 2001). In a prospective study of 11 adult patients with low-grade gliomas or benign tumors such as pituitary adenomas and meningiomas treated with fWBI, MRS detected significant decreases in both the NAA:Cr and Cho:Cr ratios starting 3 weeks after fWBI that persisted up to 6 months after fWBI in normal appearing brain parenchyma (Sundgren et al., 2009). Similar results have been obtained in several studies with glioma patients (Esteve et al., 1998; Walecki et al., 1999; Virta et al., 2000; Lee et al., 2004). Although the rodent data suggest that identifying an MRS biomarker for the onset/progression of cognitive impairment is unlikely, MRS may be still worthy of further study in humans.

## NON-INVASIVE DYNAMIC INTERACTION BIOMARKERS OF RADIATION-INDUCED COGNITIVE IMPAIRMENT

**FIGURE 4:**
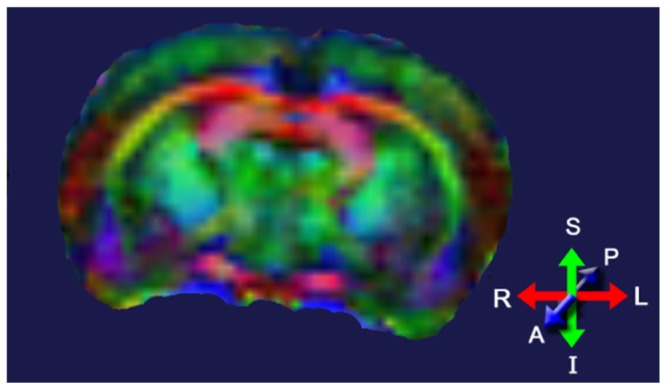
**Diffusion tensor image of a rat brain color-coded to show the predominant direction of diffusion in various brain regions; blue indicates diffusion between anterior (A) and posterior (P), red indicates flow between left (L) and right (R), and green indicates flow between superior (S) and inferior (I).** Adapted from Robbins et al. (2012).

Diffusion tensor imaging (DTI) assesses tissue microstructure by measuring the diffusion of water molecules in three-dimensional (3D) space (Le Bihan et al., 2001; Chan et al., 2009). The ability of water molecules to diffuse in brain tissue is affected predominantly by the white matter structure (i.e., the direction and compactness of the myelinated fibers in white matter tracts) and the biochemical and biophysical properties of the myelin in these tracts. Areas with little structure allow water to freely diffuse in all directions; areas with a great amount of structure will allow water to diffuse predominantly in one direction. The fractional anisotropy (FA) index is commonly used to indicate whether the water molecules in a particular region or tract are free to move in all directions (spherical diffusion) or predominantly in one direction (elliptical diffusion). FA values range from 0 to 1; low FA values indicate spherical diffusion (little structure), high FA values indicate elliptical diffusion (highly structured). DTI images are normally color-coded to indicate the primary direction of the diffusion in a particular brain region (**Figure 4**).

Relative changes in the direction of the water diffusion in 3D space after irradiation are often used to distinguish demyelination from axonal injury; this interpretation is limited to diffusion within white matter tracts. Differences in DTI parameters are also found within cortical areas and represent alterations in how water diffuses through the extracellular matrix, synaptic field, and/or lightly myelinated/unmyelinated axons. DTI indices can be compared on a voxel-by-voxel basis throughout the brain, or by summing the voxels within each ROI and comparing the results between ROIs. DTI indices can also be used to develop tractography maps of white matter bundles in the brain (Johansen-Berg and Behrens, 2006). However, the application of tractography to radiation-induced brain injury is still in its infancy.

Diffusion tensor imaging has been used to assess early white matter injury in both pediatric and adult patients treated with fWBI (Khong et al., 2006; Qiu et al., 2007; Nagesh et al., 2008; Dellani et al., 2008; Haris et al., 2008). In a recent prospective DTI study, patients with high-grade gliomas (*n* = 19), low-grade gliomas (*n* = 3), and benign tumors (*n* = 3) were imaged before, during, and after fWBI (Nagesh et al., 2008). Analyses revealed progressive DTI changes in the genu (anterior portion) and splenium (posterior portion) of the corpus callosum. During the first 3 months after fWBI, dose-dependent demyelination was detected predominantly in regions receiving high doses. However, 6 months after fWBI, this DTI detectable demyelination had spread to lower dose regions, suggesting that interventions might prevent this spread if initiated when demyelination was first detected at 3 months after fWBI (Nagesh et al., 2008).

In a cross-sectional DTI study of survivors of childhood medulloblastoma and acute lymphoblastic leukemia, FA decreases in the frontal and parietal lobes were associated with declines in intelligence quotient after adjusting for the effects of age, dose, and time after fWBI (Khong et al., 2006). The FA decreases were greater in the frontal lobes than in the parietal lobes at the same radiation dose (Qiu et al., 2007). In another study, FA values were significantly reduced in normal appearing cerebral white matter of the temporal lobe, hippocampus, and thalamus in adult survivors treated with fWBI for acute lymphoblastic leukemia (Dellani et al., 2008). In both of these studies, age-matched unirradiated controls were used as the comparison group. Given that psychiatric and health issues associated with a cancer diagnosis can influence cognition, it is imperative that neurocognitive testing as well as FA measurements be obtained prior to fWBI in future studies so that each patient can serve as their own control.

In summary, DTI is a promising non-invasive technique that is able to detect early changes in white matter integrity before radiographic evidence of radiation-induced demyelination or white matter necrosis occurs (Nagesh et al., 2008). These microanatomic changes in normal appearing white matter measure properties that likely result from dynamic interactions between irradiated oligodendrocytes, astrocytes, and neurons. However, to correlate these microanatomic changes to late delayed cognitive impairment will require that each patient undergo both DTI and cognitive testing prior to and after irradiation. Currently, there are ongoing studies that obtain DTI and cognitive impairment measurements prior to fWBI and over follow-up times as long as 18 months after fWBI in an attempt to identify DTI biomarkers which predict the onset and progression of radiation-induced cognitive impairment (Chapman et al., 2012).

**FIGURE 5:**
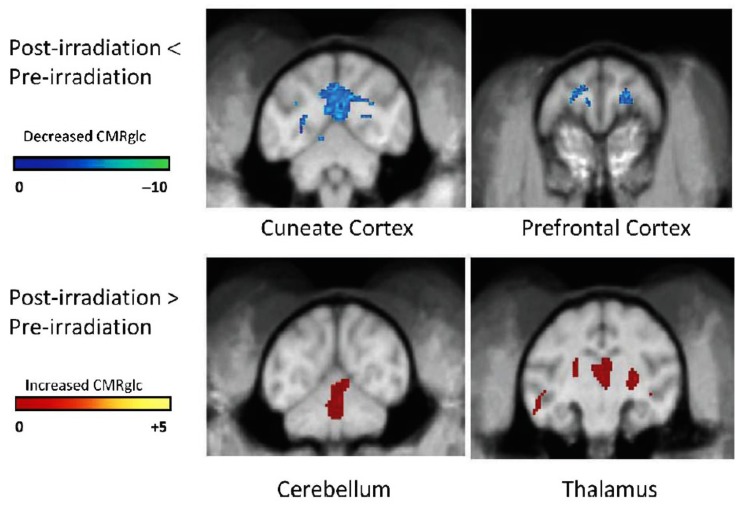
**[^**18**^F]FDG-PET scans of cerebral glucose metabolism 9 months after fWBI of young adult male non-human primates.** *Upper panel*: post-fWBI < Pre-fWBI. Blue areas in the cuneate cortex and prefrontal cortex exhibited less metabolic activity in scans obtained 9 months after fWBI than in scans obtained prior to fWBI. *Lower panel*: post-fWBI > Pre-fWBI: the red areas in the cerebellum and thalamus exhibited greater metabolic activity in scans obtained 9 months after fWBI than in scans obtained prior to fWBI. The color bar is the degree of intensity difference shown as a scale of *t* values with *P* <0.001. Adapted from Robbins et al. (2012).

Another non-invasive measure of brain function can also be obtained by quantifying the uptake of [18F]-2-deoxy-2-fluoro-D-glucose (FDG) during a cognitive task with PET. The FDG uptake in a brain region is an indicator of the level of neurosynaptic activity in that region; the neurosynaptic activity depends on the interaction among several cell types, e.g., oligodendrocytes (myelin integrity), astrocytes (glutamine/glutamate transport), and neurons (electrical pulse generation). When non-human primates (NHP) were given a total fWBI dose of 40 Gy delivered twice a week for 4 weeks, both low- and high-load cognitive function measured using a delayed match to sample (DMS) task decreased during the 12 months after fWBI; high-load function was impaired earlier than low-load function (Robbins et al., 2011). When these NHP were injected i.v. with FDG 10 min prior to a 40 min session on the DMS task and PET images acquired after completion of the DMS task, there was a decrease in FDG uptake in the cuneate and dorsal lateral prefrontal cortex and an increase in FDG uptake in the thalamus and cerebellum at 9 months after fWBI compared to the FDG uptake in these ROIs prior to fWBI (**Figure 5**). Thus, the brain regions usually involved in the DMS task did not function normally 9 months after fWBI, and increasing the activity of brain regions not usually involved in the DMS task could not adequately compensate for this deficiency. Importantly, the DMS task and the PET technique used in this NHP study can also be readily adapted for use in future clinical trials.

## PREVENTION/AMELIORATION OF RADIATION-INDUCED BRAIN INJURY

A preponderance of evidence supports the hypothesis that late radiation-induced brain injury, including cognitive impairment, is driven by acute and chronic oxidative stress and inflammatory responses (Robbins and Zhao, 2004; Zhao et al., 2007a). In general, ionizing radiation produces its biological effects by, either directly or indirectly, generating ROS that can modify a cell’s molecular or functional phenotype. An acute dose-dependent increase in ROS has been measured in cultures of astrocytes, microglia, and neurons (Ramanan et al., 2008; Robbins, unpublished data). In animals, stable end-products such as lipid peroxides and nitrotyrosine have been used to quantify the oxidative stress generated by exposure to ionizing radiation. For example, irradiating one hemisphere of 8-day-old rat brains or 10-day-old mouse brains with single 4–12 Gy doses of 4 MV X-rays resulted in an acute time-dependent increase in nitrotyrosine in both the granular cell layer of the dentate gyrus (DG) and the subventricular zone (Fukuda et al., 2004). An acute increase in lipid peroxidation was also measured in the hippocampus of adult male mice at 2 weeks after a single 10 Gy dose of WBI (Limoli et al., 2004).

### OXIDATIVE STRESS

Chronic oxidative stress is generally thought to result from an inflammatory response where irradiation activates microglia and causes immune cells to infiltrate the brain. These cells then generate ROS which in turn activate more microglia and activate more immune cells that can maintain or increase the level of oxidative stress. Interventions designed to reduce chronic oxidative stress provide an opportunity to prevent or ameliorate late radiation-induced brain injury, including cognitive impairment.

Oxidative stress is both difficult to measure and difficult to interpret, particularly in long-term studies with animals. Consequently, measures of the inflammatory response to the increase in oxidative stress after irradiation are usually used as a surrogate. In tissue culture, irradiation of mouse microglial (BV-2) cells significantly increased activation of AP-1, NF-κB, and the cAMP response element-binding protein, CREB, within the first 24 h after irradiation (Ramanan et al., 2008; Lee et al., 2010). Measurements of an acute inflammatory response have been reported in rodent models including (i) upregulation of MCP-1/CCL2 and MIP-2/CXCL2 mRNA levels (Kyrkanides et al., 2002; Kalm et al., 2009; Lee et al., 2010), (ii) increased expression of pro-inflammatory molecules such as TNFα, IL-1β, ICAM-1, and Cox-2 (Ramanan et al., 2008; Lee et al., 2010), and (iii) activation of pro-inflammatory transcription factors such as NFκB (Chiang et al., 1997; Raju et al., 1999; Kyrkanides et al., 1999, 2002; Lee et al., 2010). In a recent study, dose- and time-dependent increases in transcript levels of inflammatory cytokines, activated microglia, and activated endothelial cells were reported (Moravan et al., 2011). Finally, an acute infiltration of neutrophils and a delayed increase in T cells, MHC II-positive cells, and CD-11c-positive cells was observed in mice after single doses of ≥15 Gy (Moravan et al., 2011).

### CHRONIC INFLAMMATION

Measurements of a chronic inflammatory response to WBI and fWBI in rodent models include (i) elevation of TNFα in mouse brains up to 6 months post-irradiation (Hong et al., 1995), (ii) regionally-specific up-regulation of TNFα, and IL-1β; TNFα levels in cortex increased 57% more than in hippocampus, and IL-1β levels in hippocampus increased 126% more than in cortex (Lee et al., 2010), (iii) a marked increase in the number of activated microglia in the neurogenic zone of the DG (Monje et al., 2002), (iv) increased expression of the CCR2 receptor in the mouse subgranular zone 9 months following high-LET brain irradiation (Rola et al., 2005), and (v) persistent microglial activation in the rodent brain (Schindler et al., 2008; Ramanan et al., 2009; Conner et al., 2010). These results provide a rationale for the use of anti-inflammatory-based interventions to prevent or ameliorate late radiation-induced brain injury, including cognitive impairment.

### NEUROGENESIS

In rodents, the hippocampus plays a major role in learning, consolidation, and retrieval of information (Eichenbaum, 2001, 2004). Consequently, most rodent studies have focused on the hippocampus to investigate radiation-induced brain injury. The hippocampus consists of the DG, CA3, and CA1 regions; these regions have been implicated in both rodent and human cognition. NSCs in the DG are capable of both self-renewal and generating neurons, astrocytes, and oligodendrocytes (Palmer et al., 1997; Gage et al., 1998). Neurogenesis depends on the presence of a specific neurogenic microenvironment where endothelial cells and astrocytes can promote/regulate neurogenesis (Palmer et al., 2000; Song et al., 2002). Irradiating the hippocampus results in an increase in apoptosis in the subgranular zone of the DG (Yazlovitskaya et al., 2006), a dose-dependent increased loss of NSCs (Bellinzona et al., 1996), decreased proliferation of the surviving NSC, and decreased NSC differentiation into neurons (Snyder et al., 2001; Monje et al., 2002; Mizumatsu et al., 2003). Young adult rats irradiated with a single dose of 10 Gy produced only 3% of the new hippocampal neurons formed in unirradiated rats (Monje et al., 2002). In contrast to neurogenesis, gliogenesis appears to be preserved following irradiation (Monje et al., 2003). Interestingly, all of these phenomena can be observed after doses of ≤2 Gy that fail to produce demyelination and/or white matter necrosis.

These reductions in hippocampal neurogenesis have also been implicated in radiation-induced cognitive impairment. A decrease in hippocampal neurogenesis has been correlated with deficits in hippocampal-dependent spatial learning and memory at 3 months after a single 5 Gy dose of WBI to 21-day-old mice (Rola et al., 2004). When young adult mice received 10 Gy of focal irradiation to the hippocampus, a significant decrease in neurogenesis and cell proliferation was detected 3 months post-irradiation; this reduction correlated to a decline in cognitive function as assessed by the Barnes maze (Raber et al., 2004). Similarly, both a reduction in neurogenesis and cognitive impairment have been observed in young adult rats after fWBI (Yoneoka et al., 1999; Shi et al., 2006; Lee et al., 2012). Thus, interventions that (i) increase hippocampal neurogenesis, (ii) prevent the loss of NSCs, or (iii) replace lost NSCs after irradiation may prevent or ameliorate radiation-induced brain injury, including cognitive impairment.

## PRECLINICAL STUDIES OF THERAPEUTIC INTERVENTIONS FOR RADIATION-INDUCED BRAIN INJURY

Although the exact mechanism(s) of radiation-induced brain injury, including cognitive impairment is unclear, potential therapeutic strategies to prevent radiation-induced brain injury include ROS scavengers, anti-inflammatory agents, and NSC transplantation. ROS scavengers have received little attention because they are likely to protect brain tumors to the same extent as they protect normal brain. Thus, most of the preclinical investigations have focused on anti-inflammatory agents and fetal NSC transplantation.

**FIGURE 6:**
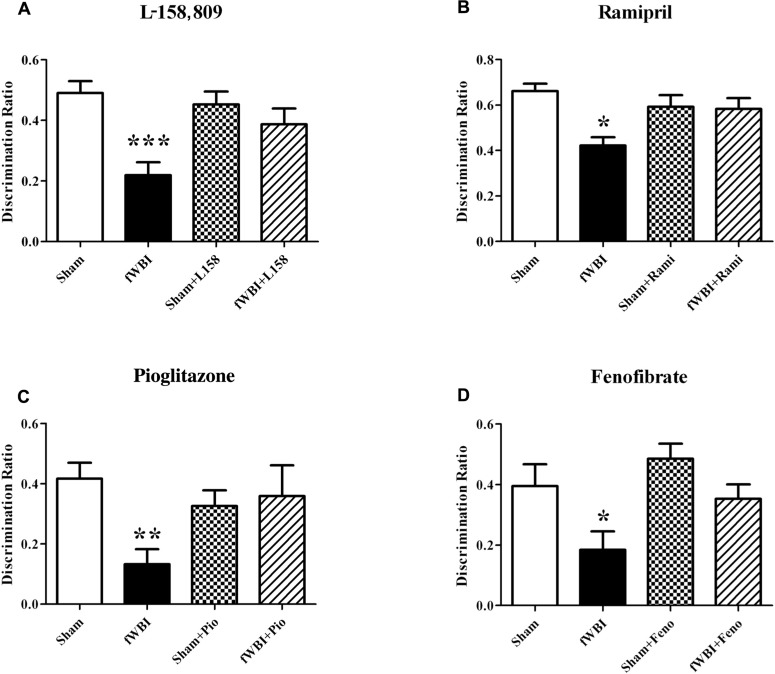
**Both RAS inhibitors and PPAR agonists prevent radiation-induced cognitive impairment in young adult male rats that received a total 40 Gy dose of fWBI delivered in 5 Gy fractions, twice/week for 4 weeks, and then tested for cognition at 6–12 months post-irradiation using the NOR task.** Rats were administered, **(A)** the ARB, L-158,809 before, during, and for 54 weeks post-fWBI; tested at 52 weeks, **(B)** the ACEI, ramipril, before, during, and for 28 weeks post-fWBI; tested at 26 weeks, **(C)** the PPARγ agonist, pioglitazone, before, during, and for 54 weeks post-fWBI; tested at 52 weeks, and **(D)** the PPARα agonist, fenofibrate, before, during, and for 29 weeks post-fWBI; tested at 26 weeks. *P <0.05, **P <0.01, ***P <0.001 compared to sham-irradiated rats.

Several rodent studies designed to prevent or ameliorate radiation-induced cognitive impairment have shown promise using anti-inflammatory peroxisome proliferator-activated (PPAR) agonists (**Figure 6**) that have been given to patients for years to treat other syndromes (Derosa, 2010; McKeage and Keating, 2011). PPARα, δ, and γ are members of the nuclear hormone receptor superfamily of ligand-activated transcription factors that heterodimerize with the retinoid X receptor to regulate gene expression (Blumberg and Evans, 1998). A growing body of evidence suggests that PPARs regulate inflammatory signaling and are neuroprotective in a variety of CNS diseases (Bright et al., 2008; Stahel et al., 2008; Ramanan et al., 2010). Administering the PPARγ agonist, pioglitazone (Pio), to young adult male rats starting 3 days prior to, during, and for 4 or 54 weeks after the completion of a total 40 Gy dose of fWBI delivered twice a week for 4 weeks, prevented the radiation-induced cognitive impairment measured 52 weeks after fWBI (**Figure 6**; Zhao et al., 2007b). However, administration of Pio for 54 weeks starting after the completion of fWBI did not significantly modulate radiation-induced cognitive impairment. Based on these data, a phase I/II trial has been initiated to determine the dose of Pio that can be given safely to brain tumor patients and obtain preliminary data on the ability of Pio to prevent/ameliorate radiation-induced cognitive impairment.

The renin–angiotensin system (RAS) has been classically viewed as a complex systemic hormonal system. More recently, several intra-organ RAS have been identified, including a brain RAS (Davisson, 2003). The brain RAS is involved in modulation of the BBB, stress, memory, and cognition (Gard, 2002; McKinley et al., 2003). Both angiotensin-converting enzyme inhibitors (ACEI) or angiotensin type-1 receptor blockers (ARB) have proven effective in treating experimental radiation nephropathy (Moulder et al., 2003) and pneumopathy (Molteni et al., 2000).

Chronic administration of the ACEI, ramipril to young adult male F344 rats 2 weeks after stereotactic irradiation of the rat brain with a single dose of 30 Gy was associated with a reduction in the severity of functional and histopathologic markers of optic neuropathy assessed 6 months post-irradiation (Kim et al., 2004). However, delaying the start of ramipril treatment to 4 weeks after irradiation resulted in a failure to reduce the severity of the radiation injury (Ryu et al., 2007). More recent studies by Jenrow et al. (2010) have shown that ramipril produced modest protection against WBI-induced decreases in neurogenesis, but did not modulate radiation-induced neuroinflammation measured as microglial activation. In contrast, a recent study found that ramipril was able to ameliorate both radiation-induced cognitive impairment (**Figure 6**) and microglial activation in rats after fWBI, but had no restorative effect on neurogenesis (Lee et al., 2012). In the Jenrow et al. (2010) study, ramipril was started 24 h after a single dose of WBI, whereas drug was administered before, during, and after fWBI in the Lee et al. study. Thus, the timing of the ramipril administration and/or the response after single or fractionated doses may explain the different results obtained in the two studies. At the present time, a phase I/II trial is being developed to determine if ramipril can prevent/ameliorate radiation-induced cognitive impairment in brain tumor patients.

Chronic administration of the ARB, L-158,809, to young adult male rats for 3 days before, during, and for 28 or 54 weeks after fWBI prevented the radiation-induced cognitive impairment observed 26 and 52 weeks post-irradiation (**Figure 6**; Robbins et al., 2009). Moreover, chronic administration of L-158,809 for 3 days before, during, and only 5 weeks post-irradiation prevented the cognitive impairment observed 26 weeks post-irradiation (Robbins et al., 2009). These radiation-induced cognitive impairments occurred without any changes in brain metabolites or gross histologic changes assessed at 28 and 54 weeks post-irradiation (Robbins et al., 2009). Thus, both PPARγ agonists and ARBs may prevent/ameliorate radiation-induced cognitive impairment when given for only a few weeks after fWBI.

In addition to drug therapeutics, there has been increased interest in the use of various stem cell therapies to restore the neurogenic niche and improve cognition. These studies are based on the rationale that radiation results in a dramatic reduction in hippocampal neurogenesis that has been linked to cognitive impairment (Raber et al., 2004; Rola et al., 2004). Voluntary running has been shown to increase neurogenesis in the rodent hippocampus with a concomitant improvement in spatial learning and memory after single WBI doses (Naylor et al., 2008; Wong-Goodrich et al., 2010). Preclinical studies have also shown that pretreatment with lithium or other Akt/glycogen synthase kinase-3β (GSK-3β) inhibitors are neuroprotective, preventing (i) apoptosis in the subgranular zone of the DG and (ii) the radiation-induced decline in hippocampal dependent memory in 1-week-old mice that received a single dose of 7 Gy WBI (Yazlovitskaya et al., 2006; Thotala et al., 2008). Direct injection of NSCs into rodent brains after WBI partially restores neurogenesis and hippocampal-dependent cognitive function (Acharya et al., 2009, 2011; Joo et al., 2012). Interestingly, these NSCs not only differentiate into neurons, but also oligodendrocytes, astrocytes, and endothelial cells that can alter the hippocampal microenvironment (Joo et al., 2012). However, the use of exercise or NSC transplantation to prevent/ameliorate radiation-induced cognitive impairment in humans will require considerably more research before it can be translated to the clinic.

### CLINICAL STUDIES OF THERAPEUTIC INTERVENTIONS FOR RADIATION-INDUCED BRAIN INJURY

One strategy for the prevention of radiation-induced cognitive impairment in the clinic involves avoidance of brain structures associated with cognitive function. Recent clinical trials have focused on avoiding the regions of adult neurogenesis, including the hippocampus and neural stem cell niche in the periventricular regions. These trials have been met with criticism because NSCs, like the stem cells found in other organ systems, are thought to be exquisitely sensitive to ionizing radiation; complete elimination of the NSCs in rodents occurs in the range of 2–6 Gy (Barani et al., 2007a,b; Gutiérrez et al., 2007). In addition, other brain regions such as the dorsal lateral prefrontal cortex play a major role in human cognition, unlike in the rodent where the hippocampus dominates. Preliminary data from the University of Wisconsin suggest that patients receiving doses ≥7.2 Gy to the bilateral hippocampi have worse cognitive function as measured by the Wechsler Memory test (Gondi et al., 2011). The RTOG is currently conducting a single arm prospective trial using hippocampal-sparing IMRT. This trial intends to enroll 100 patients and assess cognitive outcomes compared to historical controls. While technology has evolved to potentially allow for hippocampal sparing, it may be premature to conduct large-scale prospective clinical trials for hippocampal sparing when brain regions other than the hippocampus are involved in cognition, and the dose that eliminates neurogenesis in the human hippocampus is unknown.

There are no known preventive medications for radiation-induced cognitive impairment in humans, although several pharmacologic agents have been evaluated for symptomatic management. The first category of drugs assessed were the psychostimulants. There are several reports (DeLong et al., 1992; Weitzner et al., 1995; Meyers et al., 1998) using methylphenidate to treat radiation-induced fatigue and cognitive impairment. Using methylphenidate doses of 10-30mg twice daily in adults, fatigue is reduced and cognitive function is enhanced. Another class of drugs are the reversible cholinesterase inhibitors such as donepezil (Aricept^®^). The Wake Forest Community Clinical Oncology Program Research Base recently completed a clinical trial randomizing 200 brain tumor patients who survived at least 6 months after fractionated partial- or whole-brain irradiation to either placebo or donepezil 10 mg/day for 6 months. The randomized trial was based on results of a previously completed phase II open-label study where 10 mg/day of donepezil showed significant improvement in energy level, mood, and cognitive function in an identical patient population or irradiated brain tumor survivors (Shaw et al., 2006). In the phase II study, fatigue, mood, and cognition were also measured following a 6-week washout period from the discontinuation of donepezil. Worsening in all three domains was observed.

The RTOG has just completed a randomized placebo controlled trial evaluating the efficacy of memantine, an NMDA receptor antagonist that has been shown to be effective in vascular dementia. It is hypothesized that blocking this receptor blocks ischemia-induced NMDA excitation and thus, may be neuroprotective if radiation-induced ischemia occurs after fWBI. In this study, patients were treated with either memantine or placebo during and for 24 weeks after fWBI. The primary endpoint of the study involves memory deficits measured by the Hopkins Verbal Learning Test at 24 weeks. The trial has 554 patients and is now closed to accrual; to date, there are no preliminary results.

Finally, clinical trials of other potential pharmacological mediators of cognitive function are being developed based on preclinical data suggesting that anti-inflammatory agents can prevent or ameliorate radiation-induced cognitive function. A phase I/II trial of Pio given to brain tumor patients before, during, and after fWBI has been initiated, and phase I/II trials of ramipril and an ARB are being developed. Although it is simplistic to think that one approach or one pharmacological intervention will eliminate radiation-induced brain injury, including cognitive impairment for every patient whose brain is treated with ionizing radiation, it is highly likely that significant inroads will be made to prevent/ameliorate this increasingly important side effect of brain irradiation over the next decade.

## SUMMARY

Recent improvements in systemic treatments and radiation therapy techniques have resulted in over 100,000 patients in the US each year surviving long enough after fWBI to develop radiation-induced brain injury, including cognitive impairment that significantly affects their QOL. Although modern radiation therapy techniques have eliminated acute and early delayed brain injury as well as most late demyelination and white matter necrosis, dynamic interactions between multiple cell types in the brain appear to be responsible for generating late radiation-induced cognitive impairment that affects the QOL of most survivors. It is also likely that the radiation-induced cognitive impairment measured in long term survivors of SCLC, nasopharyngeal cancer, low-grade glioma, non-parenchymal tumors, primary brain tumors, and metastatic brain tumors is different because their diseases are treated differently.

Preclinical studies suggest that anti-inflammatory drugs may prevent/ameliorate radiation-induced cognitive impairment by intervening at various points in the inflammatory response to both irradiation and the presence of a brain tumor. However to date, the most effective preclinical treatments have to be given prior to, during, and continuously after irradiation. Given that ~50% of all brain tumor patients die in <6 months after fWBI, and only 50–90% of those that survive >6 months after fWBI develop radiation-induced cognitive impairment, it is imperative that non-invasive biomarkers be identified that predict who will/will not develop radiation-induced cognitive impairment, and who will/will not respond to interventions, so that treatments will be limited only to those that will ultimately benefit from them. Although the early clinical trials have had only modest success in modulating radiation-induced cognitive impairment, the future looks promising because our knowledge of how radiation-induced brain injury develops, how it can be non-invasively detected, and how it can be treated has improved considerably over the past decade.

## Conflict of Interest Statement

The authors declare that the research was conducted in the absence of any commercial or financial relationships that could be construed as a potential conflict of interest.
